# Hidden out-of-pocket spending on health: A cross-sectional study on underestimation of catastrophic health spending in Vietnam

**DOI:** 10.1016/j.puhip.2026.100817

**Published:** 2026-06-04

**Authors:** Hirotsugu Aiga, Tran Tuan Anh, Can Van Phan, Takumi Sakamoto, Marika Nomura, Hien Bui Thu, Ngoc Huy Nguyen, Khanh Minh Tran, Son Thanh Dinh Le, Hoang Van Minh

**Affiliations:** a School of Tropical Medicine and Global Health (TMGH), Nagasaki University, 1-12-4 Sakamoto, Nagasaki, 852-8523, Japan; b Institute of Tropical Medicine (NEKKEN), Nagasaki University, 1-12-4 Sakamoto, Nagasaki, 852-8523, Japan; c Human Development Department, Japan International Cooperation Agency (JICA), 3rd Floor, Nibancho Center Bldg, 5-25 Nibancho, Chiyoda-ku, Tokyo, 102-8012, Japan; d Hanoi University of Public Health, 1A Duc Thang Road, Duc Thang Ward, North Tu Liem District, Hanoi, Vietnam; e Phu Tho Provincial People's Committee, Tran Phu Str., Viet Tri City, Phu Tho province, Vietnam; f Phu Tho Provincial General Hospital, Nguyen Tat Thanh Str., Tan Dan Ward, Viet Tri City, Phu Tho province, Vietnam

**Keywords:** Catastrophic health spending, Out-of-pocket spending, Universal health coverage, Vietnam

## Abstract

**Objectives:**

To assess the extent to which excluding indirect out-of-pocket (OOP) spending leads to an underestimation of the incidence of catastrophic health spending (CHS) in Vietnam, where family members are responsible for essential in-hospital care, resulting in substantial indirect OOP spending.

**Study design:**

A cross-sectional retrospective facility-based survey.

**Methods:**

A total of 219 households with inpatient or outpatient members at Phu Tho Provincial General Hospital were interviewed using a structured questionnaire, to collect data on direct and indirect OOP spending and socioeconomic characteristics. Direct OOP spending was extracted from hospital billing records. The incidence of CHS was calculated using three thresholds: (i) spending >40% of non-food household expenditure (CHS40); (ii) spending >25% of total household expenditure (CHS25); and (iii) spending >10% of total household expenditure (CHS10).

**Results:**

Inclusion of indirect OOP spending significantly increased the incidence of CHS across the three thresholds. At the CHS40 threshold, the incidence increased from 15.1% (based on direct OOP spending only) to 31.1% after incorporating indirect OOP spending, a 2.06-fold increase. Similar trends were confirmed under the CHS25 and CHS10 thresholds. Key contributors to indirect OOP spending included health insurance premiums, meals for patients and caregivers, and transportation. Notably, 16.0% of households were categorized as experiencing CHS40 only when indirect spending was incorporated. These results highlight a substantial underestimation of financial burden under the current WHO and World Bank definition of OOP spending, which excludes indirect OOP spending.

**Conclusions:**

Excluding indirect OOP spending significantly underestimates the true incidence of CHS in Vietnam. Revising global definitions of OOP spending to include indirect OOP components may need to be considered to improve the accuracy of monitoring of financial protection. In the Vietnamese context, increasing the nurse-to-doctor ratio may help reduce indirect OOP spending and thereby the incidence of CHS. Further population-based research is needed.

**What this study adds**

■ Inclusion of indirect OOP increased CHS incidence by 1.56- 2.06 times across thresholds.
■ CHS incidence was underestimated by 11.0-19.2%, leaving many financially challenged households uncounted.

**Implications for practice**

■ Current WHO/World Bank definition of OOP fails to accurately reflect financial burden on households.
■ Vietnamese Ministry of Health should consider relaxing the nurse-to-doctor ratio to reduce reliance on informal caregiving

## 1 Introduction

Since its adoption in 2015 as one of nine targets of Sustainable Development Goal 3 (SDG 3) [1], Universal Health Coverage (UHC) has gained international attention as a key global agenda within and beyond the health sector. Financial protection is one of UHC's three dimensions. Its progress is tracked using two indicators: (i) incidence of household catastrophic health spending (CHS), and (ii) incidence of impoverishing health expenditure [2].

To determine whether a household suffers CHS, three types of thresholds have been applied. One is the threshold defined by Xu et al., in 2003, i.e. spending >40% of non-food household expenditure on health [3]. Another is the one defined by WHO and World Bank in 2015, i.e. spending >25% of total household expenditure on health [4]. In 2017, WHO and World Bank started using an additional threshold (>10% of total household expenditure) in parallel in their UHC global monitoring report [5]. While the definition of the thresholds was revised several times, the definition of out-of-pocket (OOP) spending equally necessary for calculation of incidence of CHS, has never been adequately adjusted. As the result, indirect OOP spending (e.g. transportation costs, patient feeding costs, and accommodation costs for accompanying household members) remains not incorporated into OOP spending (Table 1). Thus, indirect OOP spending can lead to underestimation of incidence of household CHE [6,7].

**Table 1:** Types of OOP spending.

Types of OOP spending	Examples	Remarks
Direct OOP spending	- Formal consultations, check-ups, examinations and treatments^a^^,^^b^ - Informal payment to health facilities or health workers (e.g. gratuity, under-the-table payment in cash or in-kind)^a^^,^^b^ - Prescribed medicines, medical orthosis, and medical equipment^a^ - Non-prescribed medicines, medical orthosis, and medical equipment^a^^,^^c^^,^^d^	Defined and recommended by WHO and World Bank^a^
Indirect OOPs spending	- Transport between home and health facilities (e.g. public transport, fuel for own vehicles/motorbikes and parking)^a^^,^^c^ - Foods for patients and accompanied caregivers (e.g. in-hospital kiosk/cafeteria, restaurant and street venders)^a^^,^^c^ - Accommodation for accompanied caregivers (e.g. hotel, guest house near health facilities)^a^^,^^c^ - Health insurance premium^e^^,^^f^^,^^g^^,^^h^^,^^i^ - Other indirect OOP spending^j^	I.e. Hidden OOP spending^d^
Total OOP spending	- All types of both direct and indirect OOP spending above^e^	I.e. direct OOP plus indirect OOP spending

a WHO and World Bank 2015 [5].

b Tanimura et al., 2014 [18].

c DiFazio and Vessey 2011 [6].

d Aiga 2015 [7].

e Goldman et al., 2018 [24].

f Nyman 1999 [25].

g Department of Health & Human Services 2015 [26].

h Bernard DS et al., 2011[27].

i Callander EJ et al., 2019[28].

j Yousefi et al., 2014 [29].

Indirect OOP spending remains excluded from the formal components of OOP spending, though WHO and World Bank admit that they are the costs related to health service utilization [4]. One possible reason why indirect OOP spending is excluded from the WHO and World Bank definitions is the limited availability of such data in general household surveys, including Living Standard Measurement Studies, which serve as key data sources for calculating the incidence of CHS in many countries. Nevertheless, informal payments (e.g. gratuities and under-the-table payments) and in-kind payments are categorized into the OOP spending components in their definition, despite equally not being included in those household surveys. This is seen as inconsistent and contradictory. In view of the significant contribution of indirect OOP spending to household CHS [[6], [7], [8], [9], [10], [11]], the current inadequate composition of OOP spending elements must not remain neglected but be proactively addressed. Otherwise, we will end up keep looking at the tip of iceberg of millions of financially challenged people [7].

In Vietnam, households are responsible for providing basic in-hospital support for inpatients, including food preparation, feeding, bathing, toileting, diaper changing, and laundry [12,13]. This includes food preparation for patients requiring alimentotherapy, which should be classified as an essential clinical intervention rather than non-clinical care. At least one household member is typically required to remain at the bedside or nearby throughout the day. Under these conditions, indirect OOP spending often places an additional financial burden on inpatient households, exacerbating hardship [7]. Non-communicable diseases (NCDs), which account for 78% of mortalities, are associated with longer hospital stays [14]. In countries like Vietnam, where indirect OOP spending is likely more common and substantial due to the epidemiological shift from infectious diseases to NCDs and population ageing, the definition of OOP spending should be urgently re-examined in light of field realities. Similar situations have been reported globally in both low- and middle-income countries (LMICs) [[15], [16], [17], [18]]).

Considering chronically high OOP share of total health expenditure since 2000s [19,20], the Government of Vietnam aimed to reduce it from 57.6% in 2010 to <40% by 2015 and beyond [21]. To achieve this target, the Vietnamese Ministry of Health has been implementing a series of reforms and adjustments in its social health insurance system. Yet, the implementation of those reforms and adjustments did not successfully lead to the reduction in OOP share below 40% by allowing it to remain at 45% as of 2019 [22]. Indirect OOP spending, synchronously with traditional direct OOP spending remaining high today, are likely to exacerbate Vietnamese people's financial hardship. Thus, this study attempts to estimate the extent to which indirect OOP spending contribute to underestimation of the incidence of CHS behind the scene, in Vietnam.

## 2 Methods

A cross-sectional study was conducted in Phu Tho province, Vietnam, to estimate the difference in incidence of CHS between three types of OOP spending definitions: (i) the combined total of direct and indirect OOP spending including health insurance premium; (ii) the combined total of direct and indirect OOP spending excluding health insurance premium and (iii) direct OOP spending only that WHO and World Bank recommend. Table 1 shows the categories of OOP spending this study employed [[5], [6], [7],^18^,[23], [24], [25], [26], [27], [28]], by their type based on the international standard [4] and earlier studies [6,7,26]. This study classified health insurance premiums as indirect OOP spending for three reasons: (i) although prepaid, premiums are restricted to health services and cannot be used for other basic needs such as food, housing, or clothing; (ii) for most people in the study area, premiums require manual payment at local social insurance offices with the option to defer or skip payment; and (iii) prior studies categorized premiums as indirect OOP spending. For these reasons, they were considered relevant to the financial burden that should be captured by OOP spending [[23], [24], [25],^27^,^28^]. To facilitate comparison with the results of previous studies, however, incidence of CHS was also calculated using the combined total of direct and indirect OOP spending excluding health insurance premiums, too.

To systematically assess the impact of including indirect OOP spending on incidence, all three CHS incidence types were calculated using three thresholds: (i) health spending >40% of non-food household expenditure (CHS40) [3]; (ii) > 25% of total household expenditure (CHS25) [4]; and (iii) > 10% of total household expenditure (CHS10) [5] (Table 2).

**Table 2:** Definitions of household catastrophic health spending (CHS).

Type	Threshold for catastrophic health expenditure	Formula
CHS40^a^	When *l* >40%, the household is identified as suffering catastrophic health expenditure.	$l=\frac{\text{OOP}\;\text{spending}\;\text{on}\;\text{health}}{\text{Total}\;\text{household}\;\text{non}-\text{food}\;\text{expenditure}}$ x 100 (%)
CHS25^b^	When *k* >25%, the household is identified as suffering catastrophic health expenditure.	$k=\frac{\text{OOP}\;\text{spending}\;\text{on}\;\text{health}}{\text{Total}\;\text{household}\;\text{expenditure}}\times 100\;\left(\%\right)$
CHS10^c^	When *k* >10%, the household is identified as suffering catastrophic health expenditure.	$k=\frac{\text{OOP}\;\text{spending}\;\text{on}\;\text{health}}{\text{Total}\;\text{household}\;\text{expenditure}}\times 100\;\left(\%\right)$

a Xu et al., 2003 [3].

b WHO and World Bank 2015 [4].

c WHO and World Bank 2017 [5].

### 2.1 Study areas

**Fig. 1:**
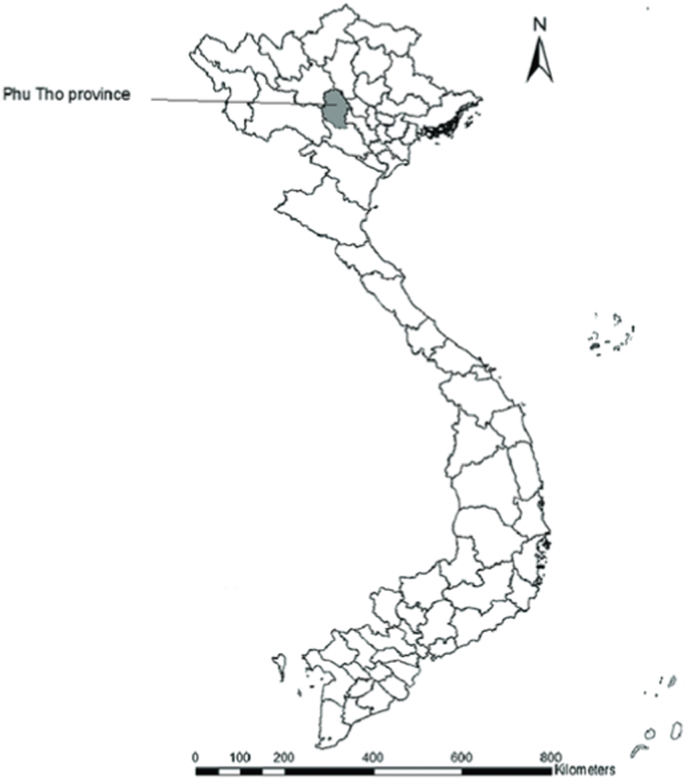
Study area for assessing hospital-based CHS.

Phu Tho is a northern Vietnamese province located 80 km from Hanoi, the national capital. It has a population of approximately 1.51 million and covers 3535 km^2^ (Fig. 1). Viet Tri, the provincial capital, has 181 thousand residents. Kinh ethnic group, Vietnam's majority, comprises 83% of the provincial population, with minorities such as Muong and Dao making up the rest [29]. Poverty rate in Phu Tho (4.1%) is close to the national average (4.2%). Given the wide provincial variation in poverty (from 0.1% in Binh Duong to 34.5% in Dien Bien), Phu Tho is socioeconomically average, with a balanced urban–rural mix [29]. Thus, Phu Tho Provincial General Hospital was selected as the study site due to the province's socioeconomic representativeness.

### 2.2 Sample size and sampling

Incidence of CHS in Vietnam was estimated at 9.4% using a threshold of OOP spending exceeding 10% of total household expenditure [30]. No prior studies assessed how the incidence changes when indirect OOP spending is included among households with inpatient or outpatient care. We therefore tentatively assumed the rate would double from 9.4% to 18.8%. Thus, sample size was calculated to detect 9.4% difference (=18.8% - 9.4%) with error = 0.05, power = 0.80, and a 5% non-response rate. The resulting sample size was 223 households. A separate comparison group was not required, as each household served as its own comparator between the three OOP spending definitions.

At Phu Tho Provincial General Hospital in Viet Tri, inpatients and outpatients were selected from patient registries in each clinical department, by using systematic random sampling. The number of patients selected per department, including the outpatient department, was proportional to that department's share of total hospital patients, following a probability-proportional-to-size (PPS) approach. Once selected, the patient's entire household was included in the study.

### 2.3 Data collection

The target group comprised households with at least one member admitted as an inpatient or visiting as an outpatient at Phu Tho Provincial General Hospital between 1 September and 7 November 2021. Data were collected not only on OOP spending but also on other household expenditures over the previous 12 months. Structured interviews were conducted from 11 November 2021 to 17 January 2022 with patients and household members responsible for financial management, including income loss, spending, income, savings, and borrowing. The 72-day gap between the hospital visit period and the interviews ensured that most sampled patients had completed treatment. Despite the ongoing COVID-19 pandemic, the hospital operated relatively normally, as data collection occurred during a plateau between the third and fourth national waves [31]. Data on direct OOP payments to the hospital were obtained from its accounting system, while other direct and indirect OOP spending and household expenditures were captured through interviews. Direct OOP payments to other facilities by patients or their household members were also recorded during interviews.

Data collected included: (i) socio-demographic and economic information for each household member; (ii) direct and indirect OOP spending per member as shown in Table 1; and (iii) other household expenditures, including food, housing, energy, education, and daily commodities. A structured questionnaire was adapted from the World Bank's Living Standard Measurement Study tool [32], translated into Vietnamese, and programmed into REDCap version 5.23.6 (Vanderbilt University, Nashville, United States), a computer-assisted personal interview (CAPI) software.

### 2.4 Data analysis

Data from the household survey were analyzed to estimate the three types of CHS incidence (CHS40, CHS25, and CHS10) based on the aforementioned three OOP spending definitions. Twelve-month timeframe was applied. McNemar's test was used to compare CHS incidence between the two OOP definitions, as the outcomes were paired for each household. To compare incidence across economic levels, households were ranked by total expenditure over the past 12 months and divided into wealth quintiles. The CHS underestimation rate was defined as the proportion of households identified as experiencing CHS only after including indirect OOP spending to t the total number of respondent households. Statistical analyses were conducted, by using *SPSS for Windows*, version 22 (IBM/SPSS Inc., Chicago, the United States).

### 2.5 Ethical considerations

The study protocol was approved by the Institutional Review Board, School of Tropical Medicine and Global Health, Nagasaki University, Japan (Ref: NU_TMGH_2021_163_1) and Hanoi University of Public Health, Vietnam (Ref: 021-357/DD-YTCC). A written informed consent to participate in the study was obtained from each patient's household member most responsible for household financing.

## 3 Results

Of 223 households sampled, four refused to participate in the study (non-response rate = 1.8%). Thus, a total of 219 households participated in the study.

### 3.1 Characteristics of participants

Table 3 presents sociodemographic and economic characteristics of participating households. Mean household size was 3.95, similar to the national mean of 3.89. Households headed by Kinh, Vietnam's majority ethnic group, accounted for 93.2%, close to the national figure of 92.2%. The proportion of household heads with higher education (college, university, or graduate level) was 22.4%, 2.6 times the national proportion of 8.76%. Despite this, health insurance enrollment rate was lower among participants (84.0%) than the national rate (90.2%). Overall, characteristics of participating households were comparable to national average, except for education level and insurance enrollment.

**Table 3:**
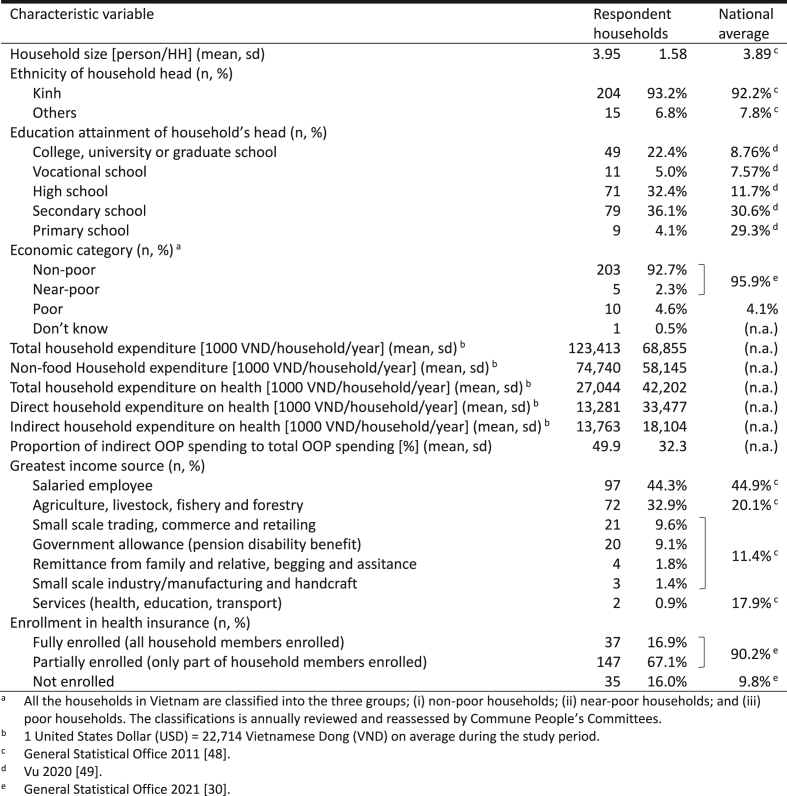
Characteristics of participating households (n = 219).

### 3.2 Incidence of household catastrophic health spending (CHS)

**Fig. 2:**
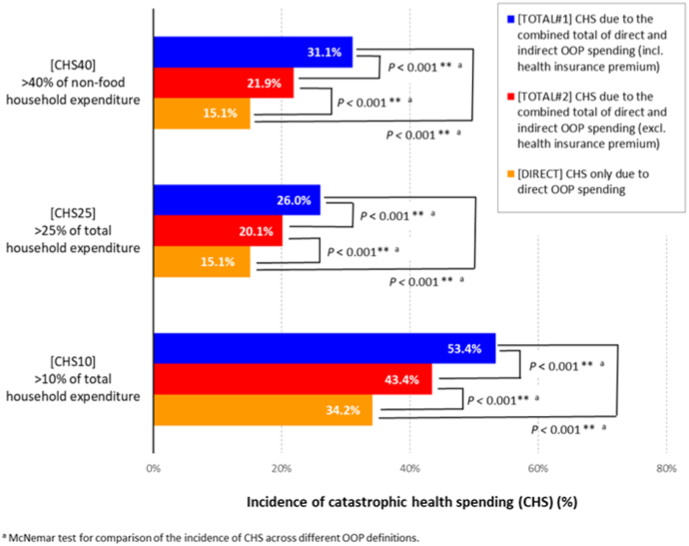
Comparison of the incidence of CHS across different OOP spending definitions.

Table 4 presents CHS incidence rates and their 95% confidence intervals by CHS threshold and expense base. This data is visualized in Fig. 2, which also compares incidence across the three different OOP spending definitions.

**Table 4:** Incidence of household catastrophic health spending (CHS) by type of OOP spending definition and by threshold (*n* = 219).

Threshold type	Incidence of household catastrophic health spending (CHS)									Rate of CHE underestimation due to exclusion of indirect OOP spending^a^			
	(a) Based on the combined total of direct and indirect OOP spending (incl. health insurance premium)			(b) Based on the combined total of direct and indirect OOP spending (excl. health insurance premium)			(c) Based only on direct OOP spending						
	*n*	(%)	95% CI	*n*	(%)	95% CI	*n*	(%)	95% CI	*n*^b^	(%)	95% CI	*P*-value^c^
CHS40	68	31.1	(25.0 – 37.6)	48	21.9	(16.6 –28.0)	33	15.1	(10.6 – 20.5)	35	16.0	(11.4 – 21.5)	<0.001
CHS25	57	26.0	(20.3 –32.4)	44	20.1	(15.0 –26.0)	33	15.1	(10.6 – 20.5)	24	11.0	(7.1 – 15.9)	<0.001
CHS10	117	53.4	(46.6 – 60.2)	95	43.4	(36.7 – 50.2)	75	34.2	(28.0 – 40.9)	42	19.2	(14.2 – 25.0)	<0.001

a The proportion of households suffering CHS only after incorporating indirect OOP spending.

b The number of households suffering CHS only after incorporating indirect OOP spending. A total of 35, 24, and 42 households were classified as incurring catastrophic health spending (CHS) at the 40%, 25%, and 10% thresholds, respectively, only when both direct and indirect out-of-pocket (OOP) spending were incorporated. These households were not considered to have CHS under the current WHO and World Bank definition, which includes only direct OOP spending. Therefore, the value in (a) does not equal the sum of (b) and (c).

c McNemar test for detecting difference in incidence rates of household CHS between total OOP and direct OOP spending bases.

For all three thresholds (CHS40, CHS25, and CHS10), the incidence of CHS based on the combined total of direct and indirect OOP spending including health insurance premiums was significantly higher (*P* < 0.001) than when based on direct OOP alone. Under CHS40, the incidence from total OOP was 31.1%, 2.06 times higher than that from direct OOP (15.1%). Similarly, for CHS25 and CHS10, incidence rates from the combined total of direct and indirect OOP spending including health insurance premiums were 26.0% and 53.4%, which were 1.72 times (=26.0%/15.1%) and 1.56 times (=53.4%/34.2%) higher than those from direct OOP, respectively. When health insurance premiums were excluded from the combined total of OOP spending in line with the WHO and World Bank definition of indirect OOP spending, the incidence of CHS at the CHS40, CHS25, and CHS10 thresholds was 21.9%, 20.1%, and 43.4%, respectively. These values remained significantly higher than those based on direct OOP spending alone (Fig. 2).

Note that, using CHS40 as the threshold, the incidence of CHS based solely on indirect OOP spending was 9.1%, indicating that indirect spending alone caused 9.1% of households to experience CHS. Similarly, indirect OOP alone led to CHS in 11.0% and 14.2% of households under CHS25 and CHS10 thresholds, respectively.

### 3.3 Rates of CHS underestimation due to the exclusion of indirect OOP spending

The right side of Table 4 presents rates of CHS underestimation resulting from excluding indirect OOP spending. These were calculated as the proportion of households having undergone CHS only after including indirect OOP, to the total number of participating households (n = 219). Underestimation was the largest with the CHS10 threshold (19.2%), followed by CHS40 (18.0%) and CHS25 (11.0%). This suggests that 32.0%, 17.4%, and 17.4% of households would have been misclassified as not experiencing CHS if only direct OOP had been considered. These households, financially affected over the past 12 months, remain overlooked and unrecognized under the traditional definition.

**Fig. 3:**
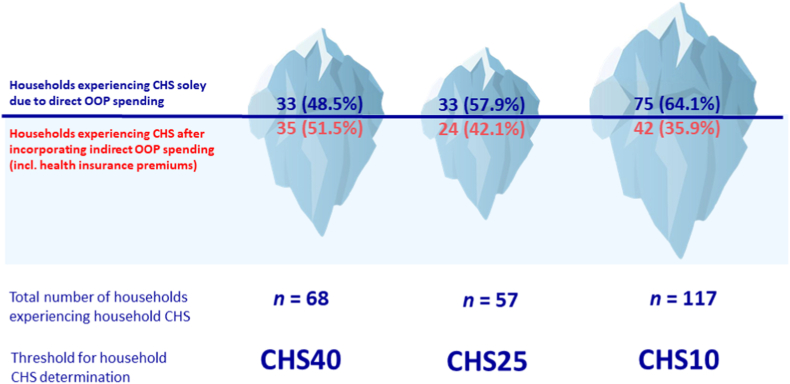
Proportion of households whose CHS would be overlooked when based solely on direct OOP spending.

Fig. 3 illustrates the proportions of households experiencing CHS only after including indirect OOP spending, as a “*bummock*', the submerged portion of an iceberg, for each threshold. Nearly half or more of total CHS cases were attributable to indirect OOP: 51.5%, 42.1%, and 35.9% under CHS40, CHS25, and CHS10, respectively. This highlights the substantial and often hidden role of indirect OOP spending.

**Fig. 4:**
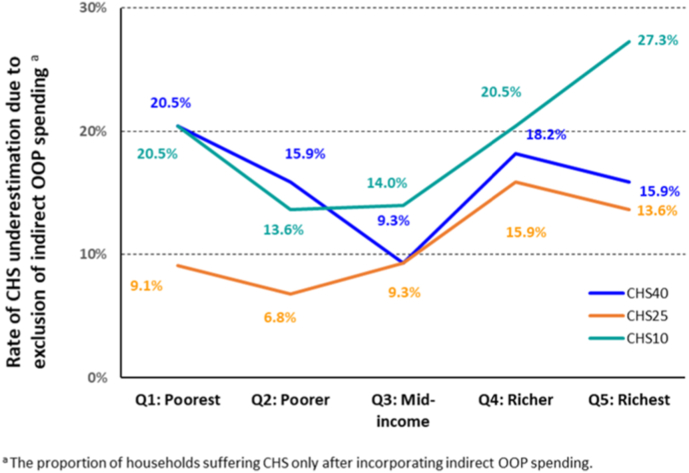
Rates of CHS underestimation due to exclusion of indirect OOP spending across wealth quintiles.

As shown in Fig. 4, rates of CHS underestimation rates vary across wealth quintiles. Notably, rates were generally higher in the richer and richest quintiles than in middle-income group. CHS10, the most sensitive threshold, produced greater fluctuations across quintiles.

### 3.4 Composition of indirect OOP spending

**Fig. 5:**
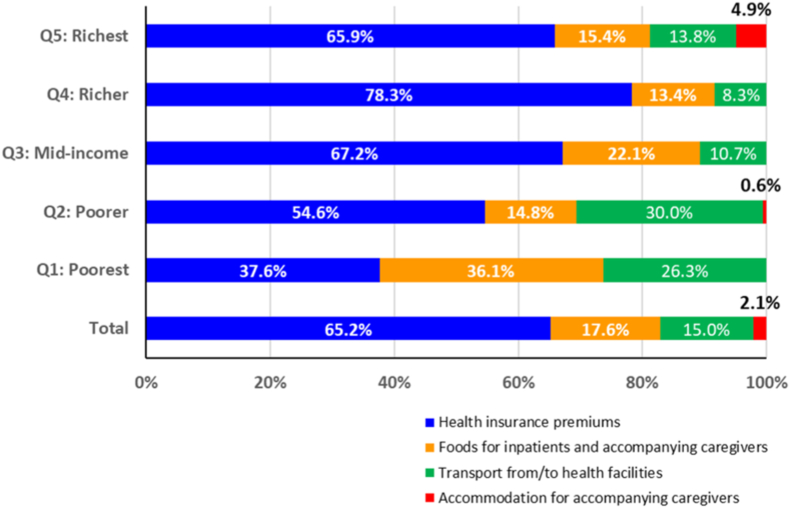
Types and composition of indirect OOP spending.

Fig. 5 shows the types and composition of indirect OOP spending by wealth quintile. Health insurance premiums accounted for the largest share (65.9%), followed by food for inpatients and caregivers (15.4%) and transportation to and from health facilities (13.8%). Accommodation for accompanying caregivers comprised a minor share (4.9%). The share of health insurance premiums, the largest component of indirect OOP spending, generally increases with wealth, from 37.6% in the poorest quintile to 67.2% in mid-income group. It then remains relatively stable in richer (78.3%) and richest (65.9%) quintiles.

## 4 Discussion

This study demonstrated that excluding indirect OOP spending substantially underestimated the incidence of CHS among households utilizing hospital services. By definition, total OOP spending, as the sum of direct and indirect components, is greater than direct OOP spending alone, in theory. Accordingly, the incidence of CHS based on total OOP was significantly higher. Increases of 2.06, 1.72, and 1.56 times across thresholds suggest substantial underestimation when indirect OOP is excluded. Moreover, indirect OOP alone led to CHS in 9.1%, 4.1%, and 17.8% of households under CHS40, CHS25, and CHS10. Excluding indirect OOP spending resulted in 19.2%, 11.0%, and 16.0% of households being overlooked, despite meeting CHS10, CHS25, and CHS40 thresholds, respectively. Notably, among those classified as experiencing CHS10, CHS25, and CHS40, 51.5%, 42.1%, and 35.9% met these thresholds only after including indirect OOP spending, respectively. These findings suggest that focusing exclusively on direct OOP spending may underestimate the financial burden associated with healthcare utilization.

While the current CHS definition based only on direct OOP spending provides a practical and internationally comparable approach, it may not fully capture the SDG and UHC principle of “leaving no one behind” [33], particularly for households experiencing substantial indirect health-related expenditures. This principle highlights the importance of identifying financially challenged households whose health-related expenditures may not be fully captured by direct OOP spending alone. At the same time, incorporating indirect OOP spending into CHS measurement presents important methodological and practical challenges. Indirect costs such as transportation, accommodation, food, and health insurance premiums may be more difficult to measure consistently than direct medical payments, particularly in large-scale household surveys. These challenges may partially explain why the current definition of OOP spending focuses on direct OOP spending at the time of service use [4]. Nevertheless, given the substantial underestimation of CHS identified in this study, the current definition may require further reassessment. Indirect OOP spending should be considered in future efforts to improve financial protection monitoring, while balancing feasibility, comparability, and data quality. If measurement difficulties justify exclusion [4], then similar challenges should also be acknowledged for informal payments, such as gratitude, in-kind, or under-the-table payments, which are difficult to capture and not recorded in facility accounts [7].

While our findings indicate substantial underestimation of CHS incidence when indirect OOP spending is excluded, caution is needed in generalizing these results, as the study focused on hospital-utilizing households in a single country. The magnitude and composition of indirect OOP spending may differ across settings depending on healthcare financing systems, service accessibility, and family caregiving practices. Accordingly, further multi-country and population-based studies are needed to assess the relevance, feasibility, and comparability of incorporating indirect OOP spending into CHS measurement frameworks and to inform potential revisions of current WHO and World Bank definitions.

The observed variation in CHS underestimation across wealth quintiles suggests important socioeconomic gradients in the financial burden of indirect OOP spending. The higher underestimation rates among richer households may reflect their greater capacity to incur indirect costs, such as transportation, food, and insurance premiums, which are not captured under the current definition. In contrast, poorer households may limit healthcare utilization or associated indirect OOP spending due to financial constraints, potentially masking their true burden. These findings highlight that excluding indirect OOP spending not only underestimates overall CHS incidence but may also distort the distribution of financial hardship across socioeconomic groups.

No previous study compared CHS incidence between direct OOP and the combined total of direct and indirect OOP spending bases. Several reported the proportion of indirect OOP to total OOP spending [[6], [7], [8], [9], [10], [11]]. Yet, these focused solely on households with patients who had specific diseases. A systematic review of 49 studies on tuberculosis patients in LMICs found that only 20% of total OOP spending was direct (e.g. consultations, tests, medicines, hospitalization), while 80% was indirect OOP spending (60% for income loss and 20% for transportation and food) [8]. This study excluded income loss from indirect OOP, as it does not represent actual expenditures. If parameters *l* and *k* in the CHS formula were adjusted to account for income loss, CHS underestimation would likely be greater. Our study included households with both inpatients and outpatients, covering chronic (e.g. tuberculosis, NCDs) and acute diseases. This broader scope likely explains the lower proportion of indirect OOP in our findings (35.4%) compared with the tuberculosis-specific review (80%) [8]. DiFazio et al. [6] synthesized findings from 15 studies in four high-income countries (Canada, Switzerland, the United Kingdom, and the United States), where indirect OOP consistently exceeded direct OOP. No study has reported this proportion for Vietnam. For example, one study in the United States found that indirect OOP represented 88% of total OOP for pediatric inpatients' households [34]. Thus, across both low- and high-income settings, indirect OOP spending often surpasses direct OOP and cannot be overlooked.

### 4.1 Psychosocial consequences of unrecognized financial burden during the COVID-19

Although this study was conducted during a relatively stable phase of the COVID-19 pandemic, the potential long-term effects of unrecognized financial burden should be considered. Financial hardship during the COVID-19 had been consistently linked to adverse mental health outcomes, including anxiety, depression, and suicidal ideation [35]. Thus, there might have been increasing utilization of mental health services. On the other hand, evidence from Vietnam suggests that income loss during the pandemic led some households to delay or forgo health service utilization to reduce OOP spending [36]. Such coping behaviors may reduce short-term costs but can worsen health and contribute to psychological distress. However, this study does not allow conclusions to be drawn regarding the extent to which the COVID-19 pandemic influenced levels of direct and indirect OOP spending.

This indicates that underestimation of financial burden is also a public health concern. Households’ financial insecurity is a known driver of psychological distress, particularly in crisis and recovery periods [37]. If indirect costs are excluded from CHS measurement, vulnerable populations at risk of both financial and mental health problems may be overlooked. Incorporating indirect costs into UHC monitoring could improve identification of at-risk groups and support more effective policy responses.

### 4.2 Nurse-to-doctor ratio in Vietnam

In this study, indirect OOP spending, including food and accommodation for accompanying caregivers, substantially increased the estimated incidence of CHS. One possible structural factor contributing to these indirect costs in Vietnam is the limited nursing workforce relative to population care needs.

In Vietnam, the proportion of deaths from NCDs increased from 78% in 2014 [14] to 81% in 2019 [38]. As NCDs increase, the demand for nursing professionals also grows [39], since NCDs are typically chronic and require longer and more frequent care than infectious diseases [40]. Thus, countries with high NCD burdens generally require a larger nursing workforce. However, Vietnam's nurse-to-doctor ratio has remained relatively low, increasing only slightly from 1.19 in 2008 to 1.34 in 2012 [41]. Several Asian countries reported higher ratios despite equal or lower contributions of NCDs to mortality: Japan (4.7; 85%), Indonesia (3.7; 76%), Korea (2.9; 78%), and India (2.0; 66%) compared with Vietnam (1.34; 81%) [42].

A persistently low nurse-to-doctor ratio may contribute to hospitals relying on household members for basic inpatient care, such as assistance with meals, hygiene, and toileting [12]. In such situations, accompanying family members may incur additional non-medical expenditures and income loss during hospitalization, which are not fully captured under conventional CHS measures based solely on direct OOP spending. Although multiple factors likely influence indirect OOP spending, the findings of this study suggest that health workforce constraints may be one contextual factor associated with increased financial burden on households. Given Vietnam's rapid epidemiologic transition over the past two decades [43], further assessment of whether the current nurse-to-doctor ratio adequately reflects evolving care needs may be needed. In ageing and increasingly NCD-prone societies, strengthening the nursing workforce may help reduce reliance on family caregivers and potentially lessen caregiver-related indirect OOP spending borne by households [21].

### 4.3 Strengths and limitations of the study

This study has several strengths. To our knowledge, this is one of the first studies to quantitatively assess the extent to which exclusion of indirect OOP spending may underestimate the incidence of CHS using multiple CHS thresholds. In addition, direct OOP spending data were obtained from hospital billing records and complemented by detailed household survey data on indirect OOP spending, which may have improved the accuracy and comprehensiveness of expenditure measurements.

This study has also several limitations. Definitions of indirect OOP spending vary across studies. Most include transportation to and from health facilities, accommodations for accompanying family members, and food for patients and caregivers. Others also include income loss [8], childcare costs incurred during caregiving [6], and health insurance premiums [44]. To improve comparability and accuracy, standardization of categories, definitions, and measurement methods is essential. Consequently, differences in the definitions and components of indirect OOP spending may limit the comparability of our findings with those reported in other studies.

Despite efforts to ensure accuracy of household survey data using a questionnaire adapted from the Vietnamese World Bank's Living Standard Measurement Study, recall bias was unavoidable. Household expenditure over the past 12 months was reported from memory. Potential measurement errors and miscategorizations of respective household expenditure items may have introduced bias. Future studies should employ prospective diary-based data collection to address this issue. CHS incidence reported here is not directly comparable with national CHS rates presented in the biennial UHC global monitoring reports [4,5,29,30], which are based on general population data. Our sample included only households with at least one hospitalized or outpatient individual. To address this, we are currently conducting a population-based survey in Phu Tho province to estimate CHS incidence from indirect OOP spending in the general population. The findings have limited generalizability, as the data were collected at a single provincial hospital and therefore reflect a facility-based sample rather than a population-based sample. Future studies across diverse settings, including multi-country and population-based designs, are needed. This study does not allow a clear interpretation of the impact of the COVID-19 pandemic on changes in health service utilization or levels of OOP spending.

### 4.4 Conclusions

This study suggests that excluding indirect OOP spending from CHS measurement may significantly underestimate the incidence of CHS. Households experiencing considerable indirect OOP spending may remain unrecognized under current CHS frameworks based solely on direct OOP spending. While the current WHO and World Bank definition of OOP spending offers a practical and internationally comparable approach for monitoring financial protection, the findings of this study indicate that indirect OOP spending may also play an important role in household financial hardship. Further multi-country and population-based studies are needed to assess whether and how indirect OOP spending can be incorporated into CHS measurement in a feasible and comparable manner. Current approaches to financial protection monitoring may benefit from broader consideration of health-related expenditures that are not fully captured by direct OOP spending alone.

## Ethics statement

The study protocol was approved by the Institutional Review Board, School of Tropical Medicine and Global Health, Nagasaki University, Japan (Ref: NU_TMGH_2021_163_1) and Hanoi University of Public Health, Vietnam (Ref: 021-357/DD-YTCC).

## Authors’ contributions

All the authors made substantial intellectual contributions to the study. HA, MN and HVM conceptualized and designed the study. TTA, CVP and HBT supervised data collection. HA and TS processed, analyzed and interpreted the data. HA drafted and finalized the manuscript. TTA, CVP, TS, MN, NHN, KMT, STDL and HVM critically commented and revised manuscript. All the authors reviewed and approved the final version of the manuscript.

Authors’ acronyms: Hirotsugu Aiga (HA); Tran Tuan Anh (TTA); Can Van Phan (CVP); Takumi Sakamoto (TS); Marika Nomura (MN); Hien Bui Thu (HBT); Ngoc Huy Nguyen (NHN); Khanh Minh Tran (KMT); Son Thanh Dinh Le (STDL); and Hoang Van Minh (HVM).

## Funding

This work was financially supported by Grant-in-Aid for Research Activity Start-up from Japan Society for the Promotion of Science (Grant No.20K23228).

## Declaration of competing interest

The authors declare that they have no known competing financial interests or personal relationships that could have appeared to influence the work reported in this paper.
